# Lymphocytic Interstitial Pneumonia Presenting With Haemoptysis and a Solitary Spiculated Pulmonary Nodule: A Case Report

**DOI:** 10.1002/rcr2.70416

**Published:** 2025-11-29

**Authors:** Xiao‐Mei Zhong, Hui‐Ming Yin, Cheng Jiang, Jie Lin, Feng‐Yang Han, Jian‐Ming Lei

**Affiliations:** ^1^ Department of Pulmonary and Critical Care Medicine The First Affiliated Hospital of Hunan University of Medicine Huaihua China; ^2^ Department of Thoracic Surgery The First Affiliated Hospital of Hunan University of Medicine Huaihua China; ^3^ Department of Cardiovascular Medicine The First Affiliated Hospital of Hunan University of Medicine Huaihua China

**Keywords:** haemoptysis, lymphocytic interstitial pneumonia, solitary pulmonary nodule

## Abstract

Lymphocytic interstitial pneumonia (LIP) is a rare disorder that typically presents with cystic changes on computed tomography. We report a distinctive case of a 53‐year‐old man with LIP whose sole presenting symptom was haemoptysis. Imaging revealed a solitary spiculated pulmonary nodule, highly suspicious for malignancy. Diagnosis was confirmed by surgical resection and histopathology, which demonstrated characteristic diffuse lymphoplasmacytic infiltration. This case underscores that LIP can masquerade radiologically as lung cancer and must be considered in the differential diagnosis of solitary pulmonary nodules, emphasising the indispensability of pathological confirmation.

## Graphical abstract

Lymphocytic interstitial pneumonia (LIP) typically shows cystic change on CT, but it can masquerade as lung cancer. We report a 53‐year‐old man with haemoptysis and a solitary spiculated left upper‐lobe nodule; surgical resection yielded a definitive diagnosis of LIP and resolution without recurrence, underscoring the need to include LIP in the differential of solitary pulmonary nodules.

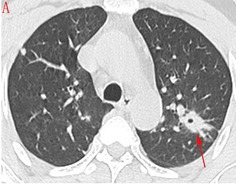

## 1 Introduction

Lymphocytic interstitial pneumonia (LIP) is a rare form of chronic interstitial pneumonia, pathologically defined by polyclonal lymphocytic infiltration of the pulmonary interstitium [1]. It is frequently associated with underlying immune dysregulation, such as Sjögren's disease or HIV infection. The typical radiological presentation consists of diffuse cystic changes and ground‐glass opacities. We report an unusual case of LIP presenting with haemoptysis and a solitary spiculated nodule, mimicking primary lung malignancy, to enhance recognition of this atypical manifestation.

## 2 Case Report

A 53‐year‐old non‐smoking man presented with a 10‐day history of cough and haemoptysis. He reported no fever, dyspnoea, chest pain, or symptoms suggestive of connective tissue disease. A prior course of cefotaxime at another institution had been ineffective. His medical history was unremarkable. On admission, vital signs were normal. Physical examination was unrevealing.

Notable investigations included a markedly elevated tuberculosis γ‐interferon release assay (T‐N 1040.65 pg/mL). Tuberculosis antibodies were weakly positive. Lymphocyte subset analysis showed a CD4/CD8 ratio of 2.1. Flow cytometry revealed the following: lymphocyte count was 782 cells/μL, total T‐lymphocyte count was 517 cells/μL, and all other parameters were within the normal range. Routine blood tests, ESR, tumour markers, autoantibody panels (including ANA, rheumatoid factor, anti‐CCP antibody, anti‐Sm antibody, anti‐dsDNA antibody, anti‐SSA/Ro‐60 antibody, anti‐SSA/Ro‐52 antibody, anti‐SSB/La antibody, anti‐U1‐RNP antibody, anti‐Scl‐70,1 antibody, anti‐Jo‐1 antibody, anti‐ribosomal P protein antibody, anti‐CENP‐B antibody, anti‐nucleosome antibody, anti‐histone antibody, anti‐PCNA antibody, anti‐M2 antibody, anti‐PM‐Scl antibody, anti‐Mi‐2 antibody, anti‐Ku antibody), MPO‐ANCA,3, PR3‐ANCA, anti‐GBM antibody, anti‐cardiolipin antibody, immunoglobulin panel (including IgA, IgG, IgE), complement C3, complement C4, and full serological screening for HIV, hepatitis, and syphilis, were all negative or within normal limits.

**FIGURE 1:**
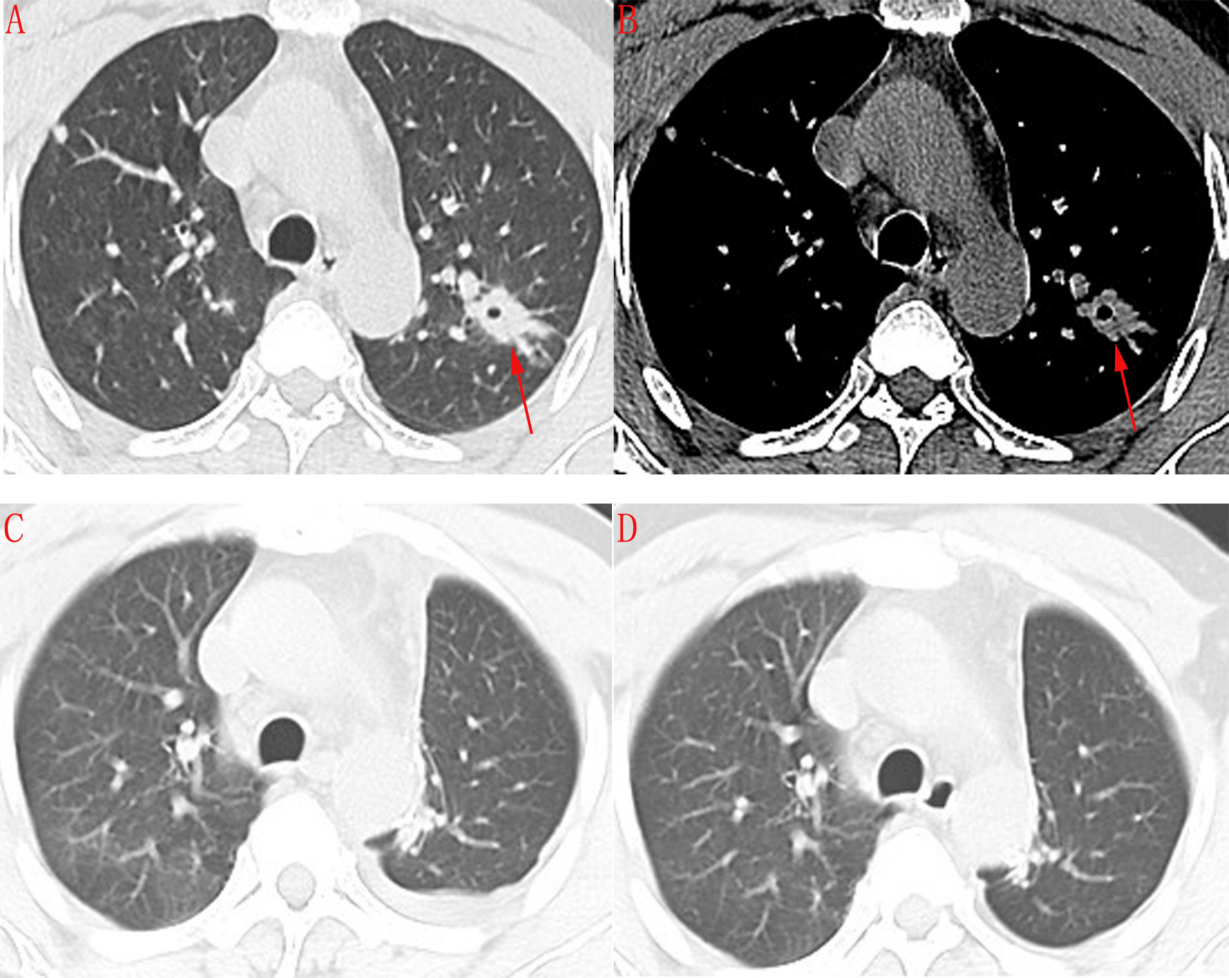
Chest CT timeline in a patient with lymphocytic interstitial pneumonia (LIP) presenting as a solitary spiculated nodule. (A, B) Baseline axial HRCT on July 7, 2022: (A) lung window shows a solitary spiculated nodule in the apicoposterior segment of the left upper lobe; (B) corresponding mediastinal window confirms a solid spiculated lesion, with internal bronchial truncation (arrow). (C) Early postoperative HRCT on July 28, 2022 shows expected postoperative change after left upper‐lobe resection with no residual lesion. (D) Three‐month follow‐up HRCT on September 5, 2022 shows no recurrence and no new nodules.

Contrast‐enhanced chest computed tomography (CT) revealed a solid, spiculated pulmonary nodule in the apicoposterior segment of the left upper lobe, with internal bronchial truncation, classified as LU‐RADS 5 (Figure 1A,B). No pulmonary cysts or ground‐glass opacities were evident. Given the high suspicion for lung cancer or tuberculosis, the patient underwent thoracoscopic left upper lobectomy. Postoperative imaging confirmed complete resection of the lesion with expected surgical changes (Figure 1C). Additional findings included a small calcified nodule in the right apex and a few non‐enlarged mediastinal lymph nodes.

**FIGURE 2:**
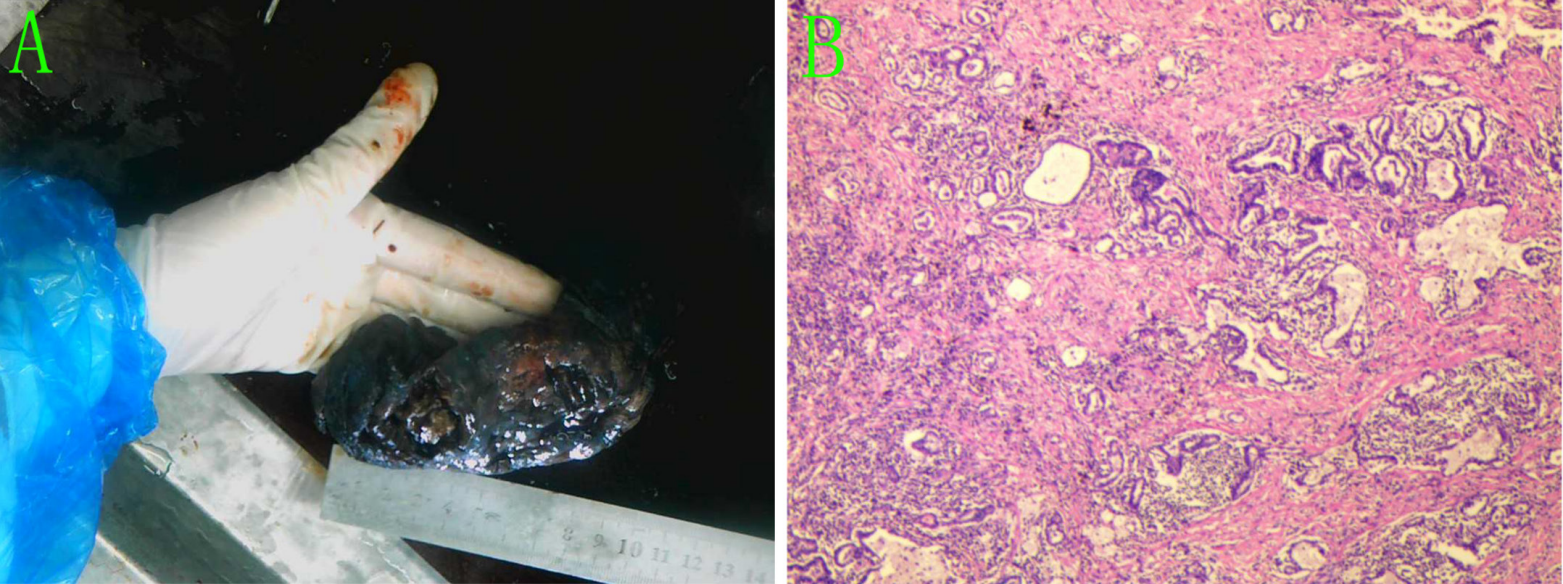
Resected specimen and histopathology of lymphocytic interstitial pneumonia. (A) Gross specimen of the resected left upper‐lobe nodule. (B) Haematoxylin–eosin section shows diffuse interstitial (septal) lymphoplasmacytic infiltration with alveolar‐septal widening, numerous lymphoid follicles, and small‐vessel proliferation with congestion/haemorrhage; focal atypical hyperplasia of alveolar epithelium is present—findings consistent with lymphocytic interstitial pneumonia. Resection margins (bronchial, vascular, and parenchymal) are negative. Immunohistochemistry: CK7+, TTF‐1+, EMA+, napsin A+, CD3+ (T‐cells), CD38+, IgG+, IgG4−; CK5/6, CK20, CEA negative; Ki‐67 = 10%. Special stains: PAS and Masson negative.

Histopathological examination of the resected specimen established the diagnosis. It showed diffuse alveolar septal expansion by a dense polyclonal infiltrate of lymphocytes and plasma cells, accompanied by prominent lymphoid follicles and focal alveolar epithelial hyperplasia. Immunohistochemical (IHC) analysis demonstrated positivity for CK7, TTF‐1, P63, EMA, CD3 (highlighting T lymphocytes), CD38, IgG, and Napsin A, while results were negative for CK5/6, CEA, IgG4, and CK20. The Ki‐67 labelling index was ~10%. Special stains (including PAS and MASSON) and comprehensive microbiological cultures for bacterial, fungal, and mycobacterial organisms were all negative (Figure 2). These features were diagnostic of LIP. The postoperative course was uneventful. The patient received no adjuvant corticosteroid therapy. Follow‐up CT at 3 months showed no evidence of recurrence (Figure 1D).

## 3 Discussion

This case illustrates a highly atypical presentation of LIP. The disease more commonly affects women and typically manifests with progressive dyspnoea and dry cough [2]. Radiologically, thin‐walled cysts and ground‐glass opacities are the hallmarks, seen in the majority of patients [3]. The occurrence of a solitary spiculated nodule, as in our patient, is exceedingly rare and poses a significant diagnostic challenge, primarily necessitating differentiation from bronchogenic carcinoma.

In this context, pathological evaluation remains the definitive diagnostic tool. The key histological features—dense polyclonal lymphoplasmacytic interstitial infiltration and lymphoid follicular hyperplasia—reliably distinguish LIP from both infections and malignant processes, particularly pulmonary lymphoma [1, 4]. In cases presenting as a solitary nodule, surgical resection serves the dual purpose of obtaining diagnostic tissue and providing curative treatment.

The optimal management for asymptomatic, localized LIP remains undefined. Our patient remained stable without immunosuppressive therapy, suggesting that a conservative approach may be sufficient in select cases. However, close long‐term follow‐up is imperative, given the documented risk of progression to pulmonary fibrosis or transformation to lymphoma [5].

In conclusion, this report highlights that LIP can radiologically mimic lung cancer as a solitary spiculated nodule. In patients with such lesions where infection and malignancy are initially suspected, LIP should be considered in the differential diagnosis. Pathological confirmation is essential, and surgical resection can be both diagnostic and therapeutic.

## Author Contributions

Conceptualization: Cheng Jiang, Jie Lin, Feng‐Yang Han. Writing – original draft preparation: Xiao‐Mei Zhong, Jian‐Ming Lei. Writing – review and editing: Xiao‐Mei Zhong, Hui‐Ming Yin. Supervision: Jian‐Ming Lei. All authors have read and agreed to the published version of the manuscript.

## Funding

The authors have nothing to report.

## Consent

The authors declare that written informed consent was obtained for the publication of this manuscript and accompanying images using the consent form provided by the Journal.

## Conflicts of Interest

The authors declare no conflicts of interest.

## Data Availability

The data that support the findings of this study are available on request from the corresponding author. The data are not publicly available due to privacy or ethical restrictions.
